# Application of adenosine 5'-triphosphate (ATP) infusions in palliative home care: design of a randomized clinical trial

**DOI:** 10.1186/1471-2458-7-4

**Published:** 2007-01-08

**Authors:** Sandra Beijer, Erik van Rossum, Pierre S Hupperets, Cor Spreeuwenberg, Marieke van den Beuken, Ron A Winkens, Lisette Ars, Ben E van den Borne, Alexander de Graeff, Pieter C Dagnelie

**Affiliations:** 1 Department of Epidemiology, Maastricht University, P.O. Box 616, 6200 MD Maastricht, The Netherlands; 2 Department of Internal Medicine/Oncology, University Hospital Maastricht, P.O. Box 5800, 6202 AZ Maastricht, The Netherlands; 3 Department of Health Care Studies, Maastricht University, P.O. Box 616, 6200 MD Maastricht, The Netherlands; 4 Pain Management and Research Centre, University Hospital Maastricht, P.O. Box 5800, 6202 AZ Maastricht, The Netherlands; 5 Department of Integrated Care, University Hospital Maastricht, P.O. Box 5800, 6202 AZ Maastricht, The Netherlands; 6 Community Care Organisation, GroenekruisDomicura Maastricht and area, Mockstraat 1, 6226 CA Maastricht, The Netherlands; 7 Department of Pulmonology, Catharina Hospital Eindhoven, P.O. Box 1350, 5602 ZA Eindhoven, The Netherlands; 8 Department of Internal Medicine/Medical Oncology, University Medical Center Utrecht, P.O. Box 85500, 3508 GA Utrecht, The Netherlands

## Abstract

**Background:**

Palliative care in cancer aims at alleviating the suffering of patients. A previous study in patients with advanced non-small-cell lung cancer showed that adenosine 5'-triphosphate (ATP) infusions had a favourable effect on fatigue, appetite, body weight, muscle strength, functional status and quality of life. The present study was designed 1. To evaluate whether ATP has favourable effects in terminally ill cancer patients, 2. To evaluate whether ATP infusions may reduce family caregiver burden and reduce the use of professional health care services, and 3. To test the feasibility of application of ATP infusions in a home care setting.

**Methods/Design:**

The study can be characterized as an open-labelled randomized controlled trial with two parallel groups. The intervention group received usual palliative care, two visits by an experienced dietician for advice, and regular ATP infusions over a period of 8 weeks. The control group received palliative care as usual and dietetic advice, but no ATP. This paper gives a description of the study design, selection of patients, interventions and outcome measures.

**Discussion:**

From April 2002 through October 2006, a total of 100 patients have been randomized. Follow-up of patients will be completed in December 2006. At the time of writing, five patients are still in follow up. Of the 95 patients who have completed the study, 69 (73%) have completed four weeks of follow-up, and 53 (56%) have completed the full eight-week study period. The first results are expected in 2007.

## Background

The World Health Organisation noted that 'the ultimate goal of palliative care is the achievement of the best quality of life for patients and their families' [1]. Complaints like progressive fatigue, deterioration in performance status, weight loss and reduced functional abilities have a substantial impact on the quality of life, and also lead to frequent and intensive use of professional health care services [2,3]. It is therefore important to develop therapies that contribute to the alleviation of these complaints in terminally ill patients.

Adenosine 5'-triphosphate (ATP) is a naturally occurring purine nucleotide which is present in every cell of the human body, well-known because of its intracellular energy-transferring role [4]. Furthermore, extracellular ATP is involved in the regulation of a variety of biological processes such as neurotransmission, muscle contraction, cardiac function, platelet function, vasodilatation, and liver glucose metabolism [4]. A previous randomized clinical trial in 58 patients with advanced non-small-cell lung cancer (NSCLC) showed that 10 intravenous 30-hour ATP infusions every 2 to 4 weeks in a clinical setting had a favourable effect on fatigue, appetite, body weight, muscle strength, functional status and quality of life [5]. Side effects (mainly chest discomfort, dyspnea and urge to take a deep breath) observed during ATP infusion were mild and disappeared rapidly after lowering the infusion rate [6].

Considering the relatively mild character of ATP therapy, application of ATP infusions in palliative home care might be a promising and relatively simple treatment to improve the quality of life and functional status of patients with advanced cancer. Based on this consideration, we initiated a study in terminally ill cancer patients, aiming:

1. To evaluate whether ATP has favourable effects in terminally ill cancer patients,

2. To evaluate whether ATP infusions may reduce family caregiver burden and reduce the use of professional health care services, and

3. To test the feasibility of application of ATP infusions in a home care setting.

In the present paper, we describe the design, selection of patients, intervention and outcome measures of this study.

## Methods/Design

### Study design and general outline

**Figure 1:**
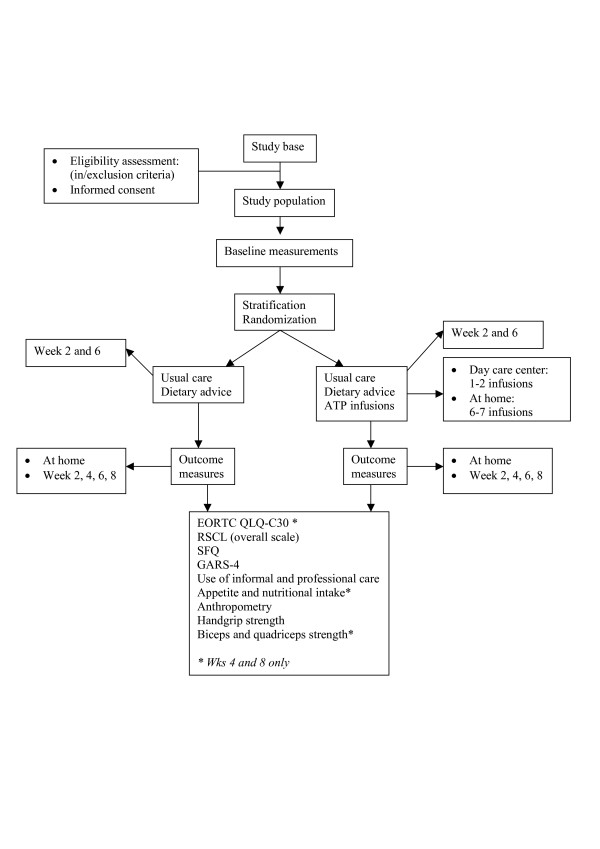
Study design.

Figure 1 displays the outline of the study design. The study can be characterized as an open-labelled randomized controlled trial with two parallel groups. Patients eligible for the study were, after stratification, randomly allocated to the intervention or control group. The intervention group received palliative care as usual and two visits by an experienced dietician for advice, and regular ATP infusions over a period of 8 weeks. The control group received palliative care as usual and dietetic advice, but no ATP. Primary and secondary outcomes were assessed at baseline and every two weeks thereafter, until eight weeks after randomization. To minimize patient burden, all outcome measurements were taken at the patients' home. Part of the data were collected with assistance from the patients' partner or family caregiver (e.g. dietary record, medication, use of professional care services). The study was approved by the Ethical Committee of the University Hospital Maastricht and Maastricht University.

### Study population

Eligible were patients with cytologically or histologically confirmed cancer, for whom medical treatment options were restricted to supportive care, who had a life expectancy < 6 months, had a World Health Organization (WHO) performance status 1 or 2, and suffered from at least one of the following complaints: fatigue, weight loss > 5% over the last 6 months, or anorexia. Patients with a WHO performance status > 2 were excluded because they were not able to attend the clinic and to undergo antropometric and muscle strength measurements. Furthermore, patients with symptomatic angina pectoris, symptomatic heart failure or any form of atrioventricular (AV) block (assessed by electrocardiogram) were excluded for safety reasons [7,8]. Other exclusion criteria were: life expectancy < 4 weeks, concurrent palliative chemotherapy, cognitive dysfunction, and other diseases hampering adequate follow up. The eligibility of patients was evaluated by a medical oncologist before inclusion. Written informed consent was obtained from all patients.

#### Patient recruitment

Patients were recruited through the Departments of Medical Oncology and Pulmonology of five hospitals in different regions in the Netherlands (Maastricht, Heerlen, Eindhoven and Utrecht). When the last line of chemotherapy had failed and medical treatment options were restricted to supportive care, the patient was informed about the study by the oncologist or pulmonologist. In addition, general practitioners in the region of Maastricht participated in patient recruitment.

### Randomisation

After baseline measurements, patients were randomly allocated to the treatment group, using random numbers generated by computer with a permuted block size of four. Before randomization participants were stratified for tumor type (colon cancer, breast cancer, and other types of cancer) and weight loss (≤ 5% in 6 months *vs*. > 5%), since weight loss is an important prognostic factor for deterioration of physical condition and quality of life [9].

### Intervention

#### General

Patients in both the experimental and control group received care as usual, i.e. patients were allowed to use and apply for all available health care services. As an additional support for patients both in the intervention and control group, all patients were visited at home by an experienced dietician in week 2 and 6 of the study. At these visits, using a standard checklist, problems and complaints were noted and followed by standard nutritional advice based on the type of complaints of the patient. For this purpose, information sheets were designed containing a standard nutritional advice for any of the following complaints: weight loss, anorexia, food aversions, nausea and vomiting, swallowing problems, dry mouth, painful mouth, diarrhoea, and obstipation. In case patients asked for more intensive dietary support or for specific nutritional supplements, they were referred to a regular dietician in hospital or home care.

#### ATP

Patients allocated to the experimental study group received weekly ATP infusions over a period of eight weeks. A longer infusion period was not considered feasible in these patients because of the expected rapidly deteriorating condition of participating patients.

In our previous study in NSCLC patients, 10 intravenous 30-hour ATP infusions were given at 2 to 4-week intervals [5]. Because of the advanced disease stage in the present study, a less intensive treatment schedule of 10 hours (range 8 – 12 hours) was chosen, in order to avoid ATP administration during the night or an overnight stay at the hospital. To increase the overall ATP dose given, ATP was administered on a weekly basis. A dose of 50 mcg/kg.min, or lower when side effects occurred, was chosen in order to maximize safety at home; in our previous study a large proportion of patients did not tolerate the dose of 75 mcg/kg.min. ATP infusions were started beginning at a dose of 20 mcg/kg.min, and were increased in steps of 10 mcg/kg.min every 10 minutes until a maximum dose of 50 mcg/kg.min, or until the maximally tolerated dose, if this was lower, had been reached. Thereafter, ATP was infused at a continuous rate. If any side effects occurred, the dose was reduced until side effects disappeared.

In the previous study ATP infusions were given in a clinical setting under strict medical supervision. Since patients in the present study were in a considerably later stage of disease, with consequently a shorter life expectancy, the majority of patients was supported by the general practitioner and were discharged from routine specialist care, with only occasional visits to the out-patient clinic, when necessary. For this reason, it was decided to basically administer most of the ATP infusions in the home setting. However, since initiation of ATP infusions under medical supervision in a clinical setting would facilitate the treatment of possible side effects, the first two ATP infusions were given at the day care center of the participating hospitals. Based on the mild character of the noted side effects during the first two infusions in the first 22 patients, an amendment was granted by the Ethical Committee during the study for administering only the first ATP infusion at the day care center, and all subsequent infusions at home.

At the end of the first infusion, the safety and tolerated dose of ATP was evaluated for each individual patient. Subsequent infusions were given at the patients' home by an experienced and trained nurse of the infusion team of the Community Care Organization. The maximally tolerated dose during the first infusion at the day care center was also the maximum dose for the next infusions at home. At home, ATP infusions also started with a dose of 20 mcg/kg.min and increased in steps of 10 mcg/kg.min every 10 minutes until the maximum tolerated dose had been reached. To provide maximal mobility a portable infusion pump was used during ATP administration. Patients and their partners were instructed extensively regarding the infusion procedures and to call the infusion team of the Community Health Care Organisation in case of side effects or other problems, such as technical problems.

#### Side effects and adverse events

Side effects of all ATP infusions, adjustments of dosage schedules, and any other problems occurring during the infusions were registered systematically on a standard form by the medical and nursing staff of the day care center of the participating hospitals, the nurses of the home infusion teams and the researcher according to WHO Common Toxity Criteria [10].

Any adverse event that occurred from baseline until one week after the last ATP infusion was reported by the investigator on the appropiate Case Report Form. Patients and their caregivers were instructed to contact the investigator if an adverse event occurred. All serious adverse events that were unexpected and assessed to be related to the study product were to be reported to the principal investigator of the study within 24 hours.

### Outcome assessment

Most of the outcome measures were adapted from the study from Agteresch et al [5].

#### Primary outcome measures

*Primary outcome measures *were quality of life, fatigue and physical restriction (see Table 1).

**Table 1:** Time schedule of outcome assessment

**Outcome measure**	**Measurement scale or specification**	**Timing (in weeks)**				
**Primary outcomes**		**0**	**2**	**4**	**6**	**8**
Quality of Life	EORTC QLQ-C30*	X		X		X
Quality of Life	RSCL, overall question **	X	X	X	X	X
Fatigue	SFQ***	X	X	X	X	X
Physical restriction	GARS-4****	X	X	X	X	X
						
**Secundary outcomes**						
Appetite	Appetite Questionnaire	X		X		X
Nutritional intake	3-Day food diary	X		X		X
Medication	Medication list	X	X	X	X	X
Body weight	Electronic weighing scale	X	X	X	X	X
Triceps skinfold		X	X	X	X	X
Arm circumference		X	X	X	X	X
Muscle strength	Biceps and quadriceps strength	X		X		X
Muscle strength	Handgrip strength	X	X	X	X	X
Use of professional care services	Diary and health care registration	X	X	X	X	X

* European Organization for Research and Treatment of Cancer Quality of Life Questionnaire-C30

** Rotterdam Symptom Checklist, overall question ("overall, how did you feel over the past week")

*** Short Fatigue Questionnaire

**** Groningen Activity Restriction Scale

*Quality of life *was assessed using the EORTC Quality of Life Questionnaire (QLQ-C30, version 3.0) [11,12]. The QLQ-C30 is a 30-item cancer-specific core questionnaire that addresses various domains of QoL. It contains five functional scales (physical functioning, role functioning, emotional functioning, cognitive functioning and social functioning), three symptom scales (fatigue, pain and nausea/vomiting), two items assessing global health and quality of life, and a number of single items addressing various symptoms (constipation, diarrhoea, dyspnea, anorexia and insomnia) and perceived financial impact. All items are scored on a 4-point Likert-type scale. The content areas covered by this questionnaire reflect the multi-dimensionality of the quality of life construct. The EORTC QLQ has been extensively used for assessing health-related quality of life in patients with a wide range of cancers. Studies in patients with advanced cancer with a short life expectancy showed that the questionnaire was well accepted, with a high completion rate and useful in detecting the effectiveness of palliative treatment over time. Results confirmed that the QLQ-C-30 is a reliable and valid measure of the quality of life in patients with advanced cancer [13,14].

In addition to the QLQ-C30, we used the overall question from the Rotterdam Symptom Checklist ("overall, how did you feel over the past week") as a general indicator of perceived QoL. The Rotterdam Symptom Checklist was originally validated in a Dutch study [15] and has since then been used in numerous studies in cancer patients.

*Fatigue *was measured by the Short Fatigue Questionnaire (SFQ). The SFQ is a reliable and simple instrument to assess bodily fatigue [16]. The questionnaire comprises 4 items. Each item is scored on a 7-point Likert scale.

*Physical restriction *was assessed by the Groningen Activity Restraint Scale (GARS-4) [17]. The GARS-4 was developed to assess disability in the domains of personal care (11 items) and domestic activities (7 items). Studies in persons receiving home care, healthy seniors, patients with recent diagnosed cancer, multiple sclerosis, or rheumatoid arthritis showed that the GARS is an easy to administer, reliable, and valid measure for assessing disability in the domains of ADL (Activities of Daily Living) and IADL (Instrumental Activities of Daily Living) [18,19].

#### Secondary outcome measures

*Secondary outcome measures *were:

- *Appetite*, assessed by four 100 mm visual analogue scales (VAS) related to hunger and satiety with words anchored at each end expressing the most positive or negative sensation. Some studies have investigated the correlation between hunger ratings and energy intake, i.e. the validity of the appetite ratings. Many of these have failed to demonstrate such a relationship, while others have found that there was an association [20,21]. Results obtained from VAS yield the most valuable information when combined with other aspects of energy balance [22].

- *Nutritional intake *was assessed by a 3-day food diary. Patients were taught to keep records, in portion sizes, of all the foods they had eaten on several days of the study. The portion sizes were described in household measures using the utensils commonly found in their homes. These household measures were quantified by volume by the investigator. In general, a 3-day record, randomized to cover seasonal and weekday variations is recommended to assess mean food consumption of a group of individuals [23]. A review about the validity of self-reported energy intake showed that energy intake derived from all methods of food recording can be an imprecise measure that is substantially under-reporting in groups including adults, children and adolescents, obese persons, athletes, military personnel and trekking explorers [24]. No data about patients with cancer are available.

- *Medication*; changes were registered during each home visit.

- *Body height*, measured to the nearest 0.1 centimeter.

- *Body weight *was measured without shoes, using an electronic weighing scale (Soehnle 7407 Translucia, Germany) to the nearest 0.05 kg.

- *Triceps skin fold thickness *was measured in duplicate with a Holtain^® ^skinfold caliper (CMS weighing equipment LTD, London UK) to calculate total body fat mass (FM) using the age- and gender-specific tables from Durnin and Womersley [25]. Fat-free mass (FFM) was calculated by subtracting fat mass from body weight.

- *Mid-upper arm circumference *of the dominant arm was measured with a flexible measuring tape, and *arm muscle area *derived using the equation: (arm circumference - π × triceps skinfold)^2^/4 π [26]. Arm muscle area gives an indication of the body's muscle mass as its main protein reserve [26].

- *Muscle strength *of two major muscle groups (i.e. elbow flexor and knee extensor muscles) were assessed at the dominant arm and leg using a hand/held Microfet2^® ^dynamometer (Biometrics Europe BV, Almere, The Netherlands). This technique has been validated in several patient groups, mostly in patients whit limited muscle strength [27-29]. The patient while sitting exerts a maximal force with the 90° flexed elbow, while the examiner pushes with the dynamometer against the patients' thumb pad until muscle strength is overcome (break test). Similarly, the patient exerts a maximal force with the 90° flexed knee, while the examiner pushes with the dynamometer against the patients' ankle until muscle strength is overcome. The strength of both muscle groups is measured twice at an interval of approximately one minute. Muscle strength is expressed in Newtonmeter (Nm) units and calculated by dividing the measured mean strength (dynamometer reading) by the distance from dynamometer position point to the medial humeral epicondylus medialis (elbow) and the medial femoral epicondylus medialis (knee).

*Handgrip strength *of the dominant hand was measured using a JAMAR hydraulic hand dynamometer (Saehan Corp. Masan, Korea). Handgrip strength is a simple, quick, easily performed, and readily available bedside test. Studies in several population groups showed strong positive correlations between handgrip strength and lean body mass [30]. Furthermore, handgrip strength is correlated with total body muscle strength [31].

In order to exclude inter-observer variability, longitudinal anthropometric and muscle strength measurements in each patient were performed by the same observer.

##### Caregiver burden

The second study objective was to evaluate whether ATP infusions could relieve family caregiver burden and reduce the use of professional health care services. Caregiver burden was measured at two levels: 1. informal care; 2. professional care. To measure caregiver burden we used the GARS-4. For each item it must be marked whether the patient, the family caregivers or health care professionals carried out the activities (not at all, partly, completely). Use of medical care services (hospital admissions, outpatient clinic visits, general practitioner contacts), and home care (both domestic and nursing care) was recorded every visit.

##### Process evaluation

The evaluation of feasibility of the ATP infusions was performed by open interviews with the staff of the day care center and the nurses of the infusion home teams after finishing patient recruitment. Aspects such as procedures concerning the transfer of ATP infusions from hospital to home, the responsibility of the general practitioner for the administration of home infusions and the technical and logistical bottlenecks were particularly evaluated. Furthermore, information about patient satisfaction regarding the safety and the burden of the ATP infusions was collected during each follow-up visit by one standard questionnaire for the patient and a second for his/her partner.

### Data management and statistical analysis

Data were entered using Microsoft Access 2000 and checked for errors by double data entry.

Comparability of baseline characteristics among the ATP and control group were assessed for age, gender, tumor type, performance status and weight loss by descriptive statistics (SPSS). Differences over time in the two groups were analyzed according to the 'intention-to-treat' principle by repeated-measures analysis of covariance with the use of the linear regression model. These analyses were performed with the SAS procedure Proc Mixed, with the use of the independence working correlation structure. Multivariate analyses were used to adjust the estimated effects for potential confounders.

#### Sample size/power calculation

Data from a previous study in NSCLC patients [5] (related to 8 weeks of follow-up) were used to calculate the sample size needed to detect clinically relevant effects of ATP infusion on the primary outcome measures fatigue and physical restriction. For both measures we assumed a mean deterioration in the control group similar to the one observed in the previous study. In the experimental group, based on an expected worse condition of patients in the present study when compared with our previous study in NSCLC patients, we used a conservative estimate. Based on our previous randomized trial with ATP infusions [5], it was calculated that a total of 60 patients would be sufficient to detect a between-group difference of 0.65 units in fatigue on a 4-point scale after 8 weeks with a power of 90% and a two-tailed alpha of 0.05. An uncertain factor in sample size calculation was the drop-out rate which was estimated to vary from 25–40%. Assuming a drop-out rate of 25% during the 8-week follow-up period, a total of 80 patients (40 patients per treatment arm) would be needed; in case of higher drop out (e.g. 40%) recruitment would be increased to 100 patients.

## Discussion

From April 2002 through October 2006, a total of 100 patients have been randomized: 10 patients in 2002, 5 in 2003, 22 in 2004, 40 in 2005, and 23 in 2006. The study was closed on October 31 2006. Follow-up of patients will be completed in December 2006. At the time of writing, five patients are still in follow up. Of the 95 patients who have completed the study, 69 (73%) have completed four weeks of follow-up, and 53 (56%) have completed the full eight-week study period.

We hope this study will demonstrate that ATP infusions leads to a reduction of frequently reported complaints in terminally ill cancer patients, for which no effective treatment is yet available. In addition, we hope to show that ATP-infusions can be administered safely in a home care setting. In case of positive findings, this trial may be a first step towards implementation of ATP infusions in integrated palliative care. The first results are expected in 2007.

## Competing interests

The author(s) declare that they have no competing interests.

## Authors' contributions

ER, PH, CS, MB, RW, LA and PD participated in the design of the study and SB, PH, MB, LA, BB, AG in the acquisition of data. SB drafted the manuscript with imput from all other authors. All authors read, revised and approved the final manuscript.

## Pre-publication history

The pre-publication history for this paper can be accessed here:


